# Quinolinic Acid: Neurotoxin or Oxidative Stress Modulator?

**DOI:** 10.3390/ijms141121328

**Published:** 2013-10-25

**Authors:** Lenka Kubicova, Franz Hadacek, Vladimir Chobot

**Affiliations:** 1 Department of Ecogenomics and Systems Biology, Division of Molecular Systems Biology, Faculty of Life Sciences, University of Vienna, Althanstrasse 14, Vienna A-1090, Austria; E-Mail: lenka.kubicova@univie.ac.at; 2 Albrecht-von-Haller Institut, Plant Biochemistry, Georg-August-Universität Göttingen, Justus-von-Liebig-Weg 11, Göttingen D-37077, Germany; E-Mail: franz.hadacek@biologie.uni-goettingen.de

**Keywords:** differential pulse voltammetry, Fenton reaction, hormesis, hydroxyl radical, inflammation, iron autoxidation, kynurenines, neuropathology, neurotoxicants, oxidative stress

## Abstract

Quinolinic acid (2,3-pyridinedicarboxylic acid, QUIN) is a well-known neurotoxin. Consequently, QUIN could produce reactive oxygen species (ROS). ROS are generated in reactions catalyzed by transition metals, especially iron (Fe). QUIN can form coordination complexes with iron. A combination of differential pulse voltammetry, deoxyribose degradation and Fe(II) autoxidation assays was used for explorating ROS formation in redox reactions that are catalyzed by iron in QUIN-Fe complexes. Differential pulse voltammetry showed an anodic shift of the iron redox potential if iron was liganded by QUIN. In the H_2_O_2_/FeCl_3_/ascorbic acid variant of the deoxyribose degradation assay, the dose-response curve was U-shaped. In the FeCl_3_/ascorbic acid variant, QUIN unambiguously showed antioxidant effects. In the Fe(II) autoxidation assay, QUIN decreased the rate of ROS production caused by Fe(II) oxidation. Our study confirms that QUIN toxicity may be caused by ROS generation via the Fenton reaction. This, however, applies only for unnaturally high concentrations that were used in attempts to provide support for the neurotoxic effect. In lower concentrations, we show that by liganding iron, QUIN affects the Fe(II)/Fe(III) ratios that are beneficial to homeostasis. Our results support the notion that redox chemistry can contribute to explaining the hormetic dose-response effects.

## 1. Introduction

Quinolinic acid (2,3-pyridinedicarboxylic acid; QUIN) is an intermediate of the kynurenine metabolic pathway of the amino acid, tryptophan. This pathway is primarily responsible for NAD(P)^+^ production [1]. The key intermediate kynurenine reacts further by forming (1) quinoline derivatives, and (2) pyridine derivatives, including QUIN, which can be metabolized further to nicotinic acid. Some organisms, e.g., Baker’s yeast (*Saccharomyces cerevisiae*), may also utilize QUIN from their cultivation medium as an extracellular precursor for NAD^+^ synthesis [2].

Several decades ago, QUIN was identified as an important endogenous neurotoxin that participates in the pathogeneses of various neurodegenerative and psychical diseases, such as Parkinson’s, Alzheimer’s and Huntington’s diseases, schizophrenia and depressions [1,3]. The physiological QUIN concentrations are below 100 nM, but increase to 10–40 μM in pathological conditions [4,5]. The direct administration of QUIN solutions into the animal brain caused extensive necrotic lesions of tissues, especially damaging of striatum and hippocampus [6], neurotransmitter deficit [7] and increased transition metal concentrations [8]. QUIN also reduced antioxidant defense efficacy in rat cortical supernatants containing mitochondria [5]. Many neurodegenerative and psychic diseases are accompanied by a brain tissue inflammation [3,9], leading to high ROS levels that can cause serious tissue destruction [10]. ROS production represents one of the proposed neurotoxicity mechanisms of QUIN [3].

QUIN may increase ROS production by over-excitation of the NMDA (*N*-methyl-d-aspartate) receptor and also directly by redox reactions of QUIN-iron complexes [3,4]. Iron is a transition metal essential for cell metabolism. Heme and non-heme iron serves as an important cofactor for many enzymes and is an essential component of mitochondrial and chloroplastic electron transport chains [11–13]; it is even an essential cofactor of QUIN biosynthesis [14]. Furthermore, “poorly liganded” non-ferritin iron may enter redox reactions that generate ROS [15–17]. Stipek *et al.* reported that low QUIN concentrations caused lipid peroxidation, depending on the presence of Fe(II) [18]. To the contrary, very high QUIN concentrations inhibited lipid peroxidation in an iron-ascorbate system [18], such as the rate of Fe(II) autoxidation, resulting in ROS production decreasing if it was liganded by QUIN [19]. However, the same coordination complexes enhanced the generation of hydroxyl radicals (^•^OH) in the presence of hydrogen peroxide (H_2_O_2_) [20], but ROS generation decreased when iron was removed from the QUIN complex by a more competitive ligand [18,21]. Various antioxidants decreased the lipid peroxidation that was induced by QUIN [22,23]. Neuroprotective effects were also shown by NMDA receptor antagonists [24].

Although the effects of QUIN-Fe complexes on ROS generation and lipid peroxidation were intensively studied, their redox properties warrant more exploration. We studied Fe(II)/Fe(III) redox cycling in QUIN and ethylenediaminetetraacetic acid (EDTA) coordination complexes in terms of ROS production in two chemical assays, deoxyribose degradation and Fe(II) autoxidation. In addition, we employed differential pulse voltammetry (DPV), which provided peak potentials of the electro-oxidation of the QUIN-Fe complexes.

The deoxyribose degradation assay was originally developed as a method to study interactions between the test compounds and hydroxyl radicals [25,26]. The hydroxyl radicals arise in the Fenton reaction (1).

(1) Fe(II)+H2O2→Fe(III)+O•H+O-H

The generated hydroxyl radicals attack 2-deoxy-d-ribose, the detection molecule. The final degradation products (thiobarbituric acid reactive species, TBARS) can be quantified photometrically as pink pigments after their reaction with thiobarbituric acid. The originally described reaction mixture contains hydrogen peroxide, Fe(III), ascorbic acid and 2-deoxy-d-ribose. Ascorbic acid reduces Fe(III) to Fe(II), the latter of which enters the Fenton reaction. Iron is added either as FeCl_3_ or in complex with ethylenediaminetetraacetic acid (EDTA). Iron ions that are added as chloride salt can form coordination complexes with the test compound; in the case of the addition of iron as a Fe-EDTA complex, this is not possible. Further modifications of this assay, when hydrogen peroxide and/or ascorbic acid are omitted, provide more extensive information about the redox chemistry of the test compounds and their complexes with iron [27].

The Fe(II) autoxidation assay informs one about the ROS generation by reduction of molecular oxygen that has diffused into the reaction mixture. In the first step, molecular oxygen is reduced by Fe(II) to superoxide anion radical (2).

(2) $$\text{Fe}(\text{II})+{\text{O}}_{2}\to \text{Fe}(\text{III})+{{\text{O}}_{2}}^{•-}$$

Two superoxide anion radicals (O_2_^•−^) dismutate to hydrogen peroxide (3), which can enter the Fenton reaction (1). 2-Deoxy-d-ribose is used as the detection molecule [28]. The assay is carried out in phosphate buffer, which enhances Fe(II) autoxidation and the Fenton reaction.

(3) $$2{{\text{O}}_{2}}^{•-}+2\;{\text{H}}^{+}\to {\text{H}}_{2}{\text{O}}_{2}+{\text{O}}_{2}$$

The rate of the iron autoxidation is influenced by the added test compound depending on its complex formation with iron [29,30]. A combination of deoxyribose degradation assay variants with voltammetry has been successfully used in the study of pro- and anti-oxidant activities of various flavonoids [31]. The effects of metal complex formation on the pro- or anti-oxidant activities cannot be predicted easily. Many complex properties, such as the spin states of the *d* electrons or steric factors, can influence the redox potential of iron and that of the ligand, both of which ultimately determine the redox chemistry of the complex [15,29].

## 2. Results and Discussion

**Figure 1:**
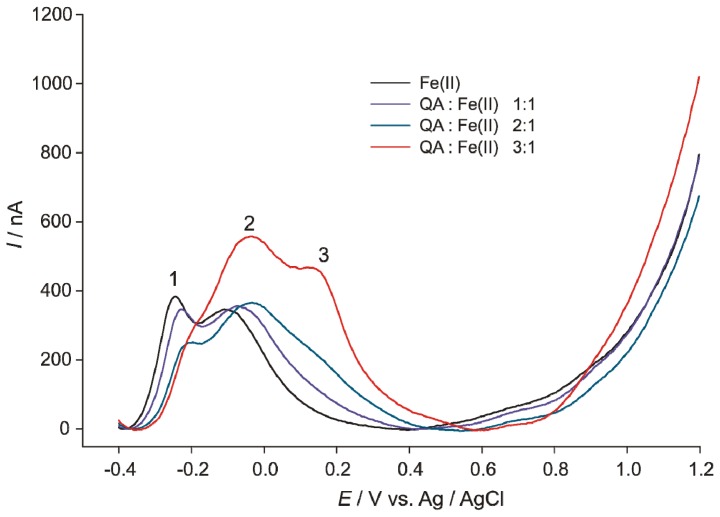
Differential pulse voltammograms of Fe(II) and 2,3-pyridinedicarboxylic acid (QUIN):Fe(II) 1:1, 2:1 and 3:1 of solutions in phosphate buffer, pH 7.4; for details, see the Experimental Section.

Differential pulse voltammograms of Fe(II), QUIN-Fe complexes and QUIN were recorded at cytoplasmic pH (Figure 1). Iron(II) ions formed various coordination complexes with the buffer component phosphate, which resulted in a broad dominant peak with two maxima at the potentials at −0.244 V (1) and −0.099 V (2). The addition of QUIN caused an anodic shift in the peak potentials. Furthermore, the peak maximum (1) became less pronounced with increasing QUIN:Fe(II) ratios and a new maximum (3) appeared. This maximum was well visible at 0.155 V in the case of a 3:1 QUIN:Fe(II) ratio. The broadness of the peak depended on the various coordination complexes of Fe(II) ions with QUIN and phosphate. High QUIN concentrations replaced phosphate in the coordination complexes to a variable extent; the characteristic orange-yellow color of QUIN-Fe(II) appeared gradually. The voltammogram reflected the influences of QUIN on the redox potential of the central atom Fe. QUIN itself, however, showed no redox activity within the measured potentials range (data not shown). The electrochemical results suggest that only QUIN-Fe complexes can account for the pro- or anti-oxidant activity, as suggested by a previous study [21].

**Figure 2:**
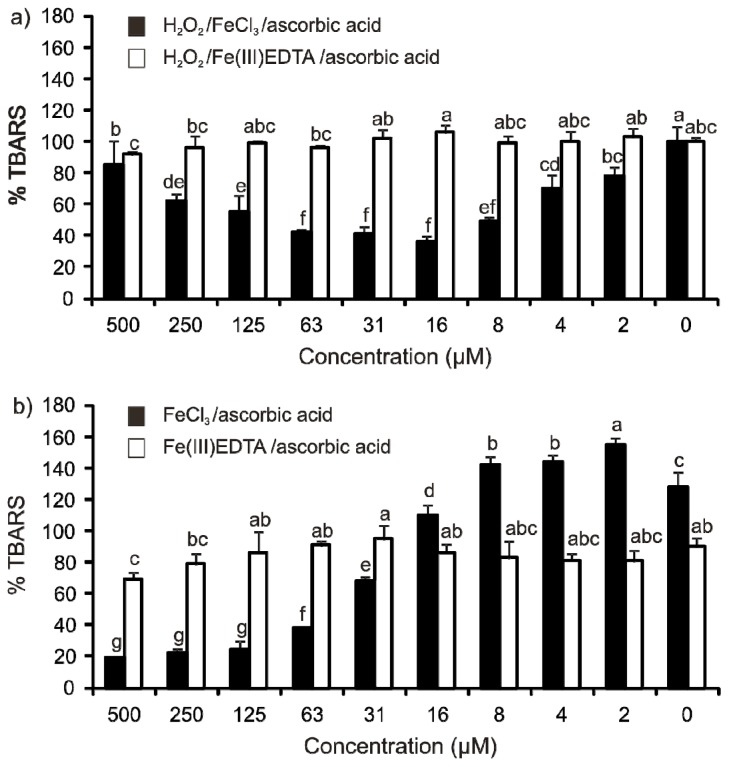
Thiobarbituric acid reactive species (TBARS) formation in (**a**) H_2_O_2_/Fe(III)/ascorbic acid (1 h incubation) and (**b**) Fe(III)/ascorbic acid (16 h incubation) variants of the deoxyribose degradation assay (100% = TBARS of the control reaction mixture of the classical variant; H_2_O_2_/FeCl_3_/ascorbic acid or H_2_O_2_/Fe(III)EDTA/ascorbic acid). EDTA, ethylenediaminetetraacetic acid. Error bars indicate the standard deviation of three replicates; letters (a, b, c, d, e, f and g) indicate different levels of significance (95% Duncan).

The deoxyribose degradation assay results also confirmed the redox activity of the QUIN-Fe complex and the inactivity of free QUIN (Figure 2). Uncomplexed QUIN was not able to start the Fenton reaction and did not affect ROS concentrations (Figure 2, white bars). Coordination complex formation was prevented by adding the iron as a Fe(III)EDTA complex. If iron was added as FeCl_3_, and if coordination complex formation with QUIN was possible, the final TBARS levels depended both on the QUIN concentrations, as well as the reaction mixture composition. This can be explained by the presence of the many different coordination complexes of QUIN and other substances with Fe ions that possess variable redox properties. The DPV showed an anodic shift of the Fe redox potential, due to the formation of QUIN-Fe complexes indicating that QUIN-Fe(II) complexes are more difficult to oxidize than phosphate-Fe(II) complexes. Despite of the anodic shift in the deoxyribose assay, QUIN-Fe(III) complexes were not reduced by hydrogen peroxide (4); this was assessed in variants that contained no ascorbic acid and in which no TBARS development could be detected, *i.e*., H_2_O_2_/FeCl_3_, FeCl_3_, H_2_O_2_/FeEDTA and FeEDTA (data not shown).

(4) $${\text{H}}_{2}{\text{O}}_{2}+\text{Fe}(\text{III})\to {{\text{O}}_{2}}^{•-}+\text{Fe}(\text{II})+2\;{\text{H}}^{+}$$

The presence of a stronger reducing agent, ascorbic acid, was required to initiate ROS generation (Figure 2). In the H_2_O_2_/FeCl_3_/ascorbic acid system, QUIN showed antioxidant effects in lower concentrations, 2–63 μM (Figure 2a, black bars). In higher concentrations, 125–500 μM, by contrast, the level of TBARS increased gradually again. The dose-response curve showed a typical U-shaped form. QUIN probably removed Fe from the competitive ligands, ascorbic acid, phosphate and 2-deoxy-d-ribose; TBARS development started to increase with higher QUIN concentrations after decreasing in lower concentrations. The inhibition of TBARS formation in the lower QUIN concentrations may be explained by an existence of various Fe coordination complexes differing in terms of ligands and, thus, central atom redox potentials. In higher QUIN concentrations, the QUIN-Fe complexes started to dominate in the reaction mixture. Iron(III) in the QUIN complex probably was more easily reduced into Fe(II) by ascorbic acid. QUIN-Fe(II) complexes were still efficient in initializing the Fenton reaction.

In the FeCl_3_/ascorbic acid system (ascorbic acid autoxidation), no pro-oxidant effects were apparent at lower QUIN concentrations, but the antioxidant effect increased with higher ones (Figure 2b, black bars). When iron was predominantly liganded by QUIN, Fe(II)/Fe(III) redox cycling was less efficient in the reduction of molecular oxygen (2) compared to the coordination complexes with ascorbic acid, which also caused higher TBARS development than the Fe(III)EDTA complex. When iron is complexed with EDTA (Figure 2b, white bars), QUIN does not interfere with ascorbic acid activities, neither in a positive nor negative fashion; similarly as in Figure 2a.

Hydrogen peroxide and ascorbic acid play crucial roles in the deoxyribose degradation assay [27]. Hydrogen peroxide addition at the beginning aims at reflecting tissue oxidative stress, as this could be caused by serious mitochondrial damage. This variant explores the capability of the test substances to interfere with ^•^OH production. Ascorbic acid is a central plant metabolite with reducing properties, but also occurs in animals tissues. It is accumulated in the mammalian brain in extracellular concentrations between 100 and 500 μM [32]. Although ascorbic acid is a well-known antioxidant, it can start ROS production, especially in the presence of iron ions, potent catalysts of electron transfer reactions [33]. ROS can participate both in beneficial, as well as pathological signal cascades in cells [10,34].

**Figure 3:**
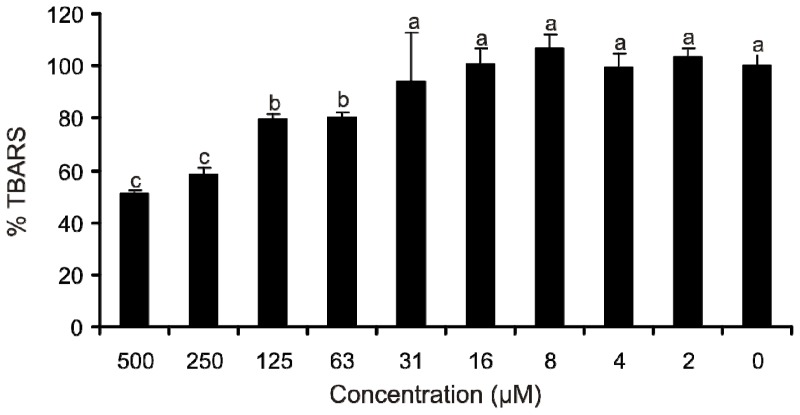
Reactive oxygen species (ROS) generation caused by QUIN in the Fe(II) autoxidation assay. ROS were detected by oxidative degradation of 2-deoxy-d-ribose and measured as thiobarbituric acid reactive species (TBARS), (100% = TBARS of the control reaction mixture without QUIN). Error bars indicate the standard deviation of three replicates; letters (a, b and c) indicate different levels of significance (95% Duncan); for details, see the Experimental Section.

The Fe(II) autoxidation assay (Figure 3) provided additional support for the proposed QUIN reaction mechanisms. QUIN as a competitive ligand removed Fe(II) ions from phosphate and decreased the autoxidation of Fe(II) in a concentration range of 63–500 μM. This concurs with the conclusion from the previous experiments and supports the notion that QUIN’s antioxidant properties are not based on scavenging, but include affecting the Fe(II)/Fe(III) redox cycling in the reaction mixture.

Here, we present a hypothetical reaction model that offers explanations for the controversial activity reports for QUIN. The results showed that QUIN can act as an antioxidant and pro-oxidant, depending on its concentration and the chemical milieu. QUIN-Fe complexes can sustain progressive neurodegenerative processes [3], especially when high ROS levels are present already. Müller *et al.* studied a possible neuroprotective mechanism of acyclovir, a nucleoside analogue and alternative iron ligand, used for the treatment of herpes simplex encephalitis [21]. Acyclovir addition decreased the production of superoxide anion radicals and inhibited lipid peroxidation by removing Fe from its complexes with QUIN. This points to the fact that the ligand identity has a substantial effect in the development of diseases, in which ROS development represents an important component.

Furthermore, our experiments also indicate that QUIN may contribute to redox homeostasis maintenance in the brain by keeping Fe(II)/Fe(III) equilibrium in a concentration- and milieu-dependent way. A combination of high ROS and QUIN concentrations can cause toxic effects that are harmful to the affected cells, but that might prime neighboring ones, especially if strong pervasive reducing agents, such as ascorbic acid, are present. Generally, the higher QUIN concentrations in patient brains suffering from neurodegenerative or psychical diseases may represent more the consequence of the oxidative stress in the affected tissue than its reason. The first experiments with QUIN used unnaturally high concentrations that were injected directly into the brain. The thus applied mechanical damage caused elevated ROS levels that were enhanced further by the injected QUIN solution, a scenario shown by Figure 2a (black bars). The endogenous concentrations of QUIN are several magnitudes lower than those used in the neuropathological models of rodents or primates *in vivo* [3].

## 3. Experimental Section

### 3.1. Chemicals

Hydrogen peroxide and 2-deoxy-d-ribose were obtained from Fluka (Buchs, Switzerland). All other chemicals used were purchased from Sigma-Aldrich (Schnelldorf, Germany). Water had Milli-Q quality.

### 3.2. Differential Pulse Voltammetry

Voltammetric curves were recorded in a three-electrode system, μAutolab PGSTAT type III (EcoChemie Inc., Utrecht, Netherlands). The working electrode was a glassy carbon electrode of 3-mm diameter; an Ag/AgCl (saturated KCl) electrode was used as reference and a platinum wire as a counter electrode. The glassy carbon electrode was washed with water and then polished by aluminum oxide powder (0.3 μm of grain size) before every measurement. The effective scan rate of the voltammetry was 21 mV s^−1^; the step potential was 5.25 mV, the modulation amplitude, 25 mV, modulation time, 50 ms, and the interval time was 250 ms. The scan potential was from −0.400 to +1.200 V. FeSO_4_ was dissolved in degassed water at a concentration 10 mM. Quinolinic acid was dissolved in degassed buffer (0.1 M phosphate buffer, pH 7.4). The ionic strength of the buffer was 1 M, and it was adjusted by K_2_SO_4_. The sample for the analysis was prepared by mixing 1 mL of aqueous FeSO_4_ solution with 9 mL of the degassed buffer or buffer quinolinic acid solution. The final concentrations of quinolinic acid were 1, 2 and 3 mM. The samples for the electrochemical measurements of quinolinic acid were prepared by mixing 9 mL of the buffer solution of the QUIN with 1 mL of water. The electrolyte solutions were degassed by argon for 10 min, and measurements were carried out under argon atmosphere at a room temperature.

### 3.3. Deoxyribose Degradation Assay Variants

The deoxyribose degradation assay and the various variants follow published procedures [27]. Quinolinic acid was dissolved in aqueous KH_2_PO_4_/KOH buffer solution (30 mM, pH 7.4) and diluted serially (2–500 μM); to 125 μL of this solution, 25 μL of a 10.4 mM 2-deoxy-d-ribose solution in the same buffer system and 50 μL of FeCl_3_ (FeCl_3_ variant) or Fe(III)EDTA (Fe(III)EDTA variant) solution (50 μM) were added. The complex of Fe(III) with EDTA was prepared separately; the 104 μM EDTA solution in the buffer was premixed with the aqueous 100 μM FeCl_3_ solution (1:1 *v*/*v*). Further, 25 μL of 10.0 mM aqueous solution H_2_O_2_ and 25 μL of 1.0 mM ascorbic acid in the buffer were added to start the Fenton reaction in the H_2_O_2_/Fe(III)/ascorbic acid reaction mixture. In the other deoxyribose degradation assays systems, H_2_O_2_ or ascorbic acid was replaced by the same volume of water or buffer, respectively. Thiobarbituric acid reactive species (TBARS) were determined photometrically at 532 nm after reaction with thiobarbituric acid and subsequent extraction of the pink pigment with 1-butanol. The H_2_O_2_/Fe(III)/ascorbic acid reaction mixture served as positive control, represented 100% TBARS detection in all variants and also served as the comparative standard for each experiment. Blanks contained the full reaction mixtures, except for 2-deoxy-d-ribose, and were determined in each experiment. Experiments were performed in triplicate. The temperature during incubation was 27 °C. Variants containing H_2_O_2_ were evaluated after 1 h; variants without H_2_O_2_ were evaluated after 16 h incubation.

### 3.4. Fe(II) Autoxidation Assay

Quinolinic acid was dissolved in aqueous KH_2_PO_4_/KOH buffer solution (30 mM, pH 7.4) and diluted serially (2–500 μM); to 125 μL of this solution, 25 μL of a 52 mM 2-deoxy-d-ribose solution in the same buffer system, 50 μL of the buffer and 50 μL of degassed aqueous FeSO_4_ solution (50 μM) were added. Blanks contained the full reaction mixtures, except for 2-deoxy-d-ribose. Standard 1.5 mL sample vials (La-Pha-Pack, Werner Reifferscheidt GmbH, Langerwehe, Germany) were used as reaction vials. The mixture was incubated at 27 °C for 16 h. Thereafter, 250 μL of 1.0% thiobarbituric acid dissolved in 3% trichloroacetic acid (*w*/*v*) was added to each vial to detect TBARS. The vials were heated in a water bath at 85 °C for 30 min. The reaction was stopped by transferring the vials into an ice water bath for 3 min. To extract the TBARS, 600 μL of 1-butanol was added, and the mixture was rigorously vortexed. The butanol layers of the vials, each 350 μL, were pipetted into flat bottomed 96-well plates (Greiner, Kremsmünster, Austria), and the absorbance was determined with a microplate reader (Tecan Infinite M200, Männedorf, Switzerland) at 532 nm. Experiments were performed in triplicate. Reaction mixtures lacking the test compound served as the positive control (100% TBARS). The phosphate buffer and water, which were used as solvents for QUIN or FeSO_4_, were degassed by argon for 10 min at least.

### 3.5. Statistical Analysis

Statgraphics Centurion XVI (Statistical Graphics Corp., Rockville, MD, USA) was used to perform analyses of variance (ANOVA), employing Duncan’s 95% multiple range *post hoc* test.

## 4. Conclusions

A combination of high ROS levels and high QUIN concentrations cause pro-oxidant and cytotoxic effects that may be responsible for the neurotoxicity assigned to QUIN and can stimulate the pathogenesis of many neurodegenerative and psychical diseases. Our study confirms that QUIN toxicity is caused by ROS generation via the Fenton reaction. This, however, applies only for unnaturally high concentrations that were used in studies attempting to provide support for the neurotoxic effect. In lower concentrations, however, we provide experimental evidence suggesting that by liganding iron, QUIN affects the Fe(II)/Fe(III) ratios in the cell, such that redox homeostasis can prevail. Our results also support the notion that redox chemistry can contribute to explaining the hormetic dose-response effects [35].

## References

[1] Zádori D., Klivényi P., Vámos E., Fülöp F., Toldi J., Vécsei L. (2009). Kynurenines in chronic neurodegenerative disorders: Future therapeutic strategies. J. Neural. Transm.

[2] Ohashi K., Kawai S., Murata K. (2013). Secretion of quinolinic acid, an intermediate in the kynurenine pathway, for utilization in NAD^+^ biosynthesis in the yeast*Saccharomyces cerevisiae*. Eukaryot. Cell.

[3] Schwarcz R., Bruno J.P., Muchowski P.J., Wu H.Q. (2012). Kynurenines in the mammalian brain: When physiology meets pathology. Nat. Rev. Neurosci.

[4] Guillemin G.J. (2012). Quinolinic acid, the inescapable neurotoxin. FEBS J.

[5] Leipnitz G., Schumacher C., Scussiato K., Dalcin K.B., Wannmacher C.M.D., Wyse A.T.D., Dutra C.S., Wajner M., Latini A. (2005). Quinolinic acid reduces the antioxidant defenses in cerebral cortex of young rats. Int. J. Dev. Neurosci.

[6] Vezzani A., Forloni G.L., Serafini R., Rizzi M., Samanin R. (1991). Neurodegenerative effects induced by chronic infusion of quinolinic acid in rat striatum and hippocampus. Eur. J. Neurosci.

[7] Nemeth H., Toldi J., Vecsei L. (2006). Kynurenines, Parkinson’s disease and other neurodegenerative disorders: Preclinical and clinical studies. J. Neural. Transm.

[8] Santamaria A., Rios C., Perez P., Flores A., Galvan-Arzate S., Osorio-Rico L., Solis F. (1996). Quinolinic acid neurotoxicity: *In vivo* increased copper and manganese content in rat corpus striatum after quinolinate intrastriatal injection. Toxicol. Lett.

[9] Felger J.C., Lotrich F.E. (2013). Inflammatory cytokines in depression: Neurological mechanisms and therapeutic implications. Neuroscience.

[10] Graves D.B. (2012). The emerging role of reactive oxygen and nitrogen species in redox biology and some implications for plasma applications to medicine and biology. J. Phys. D Appl. Phys.

[11] Andreini C., Bertini I., Cavallaro G., Holliday G.L., Thornton J.M. (2008). Metal ions in biological catalysis: From enzyme databases to general principles. J. Biol. Inorg. Chem.

[12] Atkinson A., Winge D.R. (2009). Metal acquisition and availability in the mitochondria. Chem. Rev.

[13] Vigani G., Zocchi G., Bashir K., Philippar K., Briat J.F. (2013). Signals from chloroplasts and mitochondria for iron homeostasis regulation. Trends Plant Sci.

[14] Stachowski E.K., Schwarcz R. (2012). Regulation of quinolinic acid neosynthesis in mouse, rat and human brain by iron and iron chelators *in vitro*. J. Neural. Transm..

[15] Welch K.D., Davis T.Z., Van Eden M.E., Aust S.D. (2002). Deleterious iron-mediated oxidation of biomolecules. Free Radic. Biol. Med.

[16] Kell D.B. (2010). Towards a unifying, systems biology understanding of large-scale cellular death and destruction caused by poorly liganded iron: Parkinson’s, Huntington’s, Alzheimer’s, prions, bactericides, chemical toxicology and others as examples. Arch. Toxicol.

[17] Rivera-Mancia S., Perez-Neri I., Rios C., Tristan-Lopez L., Rivera-Espinosa L., Montes S. (2010). The transition metals copper and iron in neurodegenerative diseases. Chem.-Biol. Interact.

[18] Stipek S., Stastny F., Platenik J., Crkovska J., Zima T. (1997). The effect of quinolinate on rat brain lipid peroxidation is dependent on iron. Neurochem. Int.

[19] Platenik J., Stopka P., Vejrazka M., Stipek S. (2001). Quinolinic acid-iron(II) complexes: Slow autoxidation, but enhanced hydroxyl radical production in the Fenton reaction. Free Radic. Res.

[20] Iwahashi H., Kawamori H., Fukushima K. (1999). Quinolinic acid, α-picolinic acid, fusaric acid, and 2,6-pyridinedicarboxylic acid enhance the Fenton reaction in phosphate buffer. Chem.-Biol. Interact.

[21] Muller A.C., Dairam A., Limson J.L., Daya S. (2007). Mechanisms by which acyclovir reduces the oxidative neurotoxicity and biosynthesis of quinolinic acid. Life Sci.

[22] Sadeghnia H.R., Kamkar M., Assadpour E., Boroushaki M.T., Ghorbani A. (2013). Protective effect of safranal, a constituent of *Crocus sativus*, on quinolinic acid-induced oxidative damage in rat hippocampus. Iran. J. Basic Med. Sci.

[23] Perez-Severiano F., Rodriguez-Perez M., Pedraza-Chaverri J., Maldonado P.D., Medina-Campos O.N., Ortiz-Plata A., Sanchez-Garcia A., Villeda-Hernandez J., Galvan-Arzate S., Aguilera P. (2004). S-Allylcysteine, a garlic-derived antioxidant, ameliorates quinolinic acid-induced neurotoxicity and oxidative damage in rats. Neurochem. Int.

[24] Stone T.W. (2000). Development and therapeutic potential of kynurenic acid and kynurenine derivatives for neuroprotection. Trends Pharmacol. Sci.

[25] Halliwell B., Gutteridge J.M.C., Aruoma O.I. (1987). The deoxyribose method—A simple test-tube assay for determination of rate constants for reactions of hydroxyl radicals. Anal. Biochem.

[26] Aruoma O.I., Grootveld M., Halliwell B. (1987). The role of iron in ascorbate-dependent deoxyribose degradation—Evidence consistent with a site-specific hydroxyl radical generation caused by iron ions bound to deoxyribose molecule. J. Inorg. Biochem.

[27] Chobot V. (2010). Simultaneous detection of pro- and antioxidative effects in the variants of the deoxyribose degradation assay. J. Agric. Food Chem.

[28] Gutteridge J.M.C. (1984). Reactivity of hydroxyl and hydroxyl-like radicals discriminated by release of thiobarbituric acid-reactive material from deoxy sugars, nucleosides and benzoate. Biochem. J.

[29] Pierre J.L., Fontecave M. (1999). Iron and activated oxygen species in biology: The basic chemistry. Biometals.

[30] Welch K.D., Davis T.Z., Aust S.D. (2002). Iron autoxidation and free radical generation: Effects of buffers, ligands, and chelators. Arch. Biochem. Biophys.

[31] Chobot V., Kubicova L., Bachmann G., Hadacek F. (2013). Versatile redox chemistry complicates antioxidant capacity assessment: Flavonoids as milieu-dependent anti- and pro-oxidants. Int. J. Mol. Sci.

[32] Grunewald R.A. (1993). Ascorbic acid in the brain. Brain Res. Rev.

[33] Buettner G.R. (1988). In the absence of catalytic metals ascorbate does not autoxidize at pH 7: Ascorbate as a test for catalytic metals. J. Biochem. Biophys. Methods.

[34] Valko M., Leibfritz D., Moncol J., Cronin M.T.D., Mazur M., Telser J. (2007). Free radicals and antioxidants in normal physiological functions and human disease. Int. J. Biochem. Cell Biol.

[35] Mao L., Franke J. Hormesis in aging and neurodegeneration—A prodigy awaiting dissection.

